# Supplementary material for: Comparison of 6-week PMTCT outcomes for HIV-exposed and HIV-unexposed infants in the era of lifelong ART: Results from an observational prospective cohort study
Source: PLoS One. 2019 Dec 26;14(12):e0226339. doi: 10.1371/journal.pone.0226339 (PMC6932788; doi:10.1371/journal.pone.0226339)
Supplement: Attachment — Submitted filename: Response to Reviewers comment-Birth Outcomes in Lesotho_2.docx [file pone.0226339.s003.docx]

**Response to PLOS One**

Dear Academic Editor PLOS ONE,

Thank you for the opportunity to revise our manuscript.

The reviewers’ comments were much appreciated. We have addressed the comments in the revised manuscript. In addition, specific responses to the reviewers’ questions follow each comment below.

PONE-D-19-18206
**Comparison of 6-week PMTCT outcomes for HIV-exposed and HIV-unexposed infants in the era of lifelong ART: results from an observational prospective cohort study**

General Comments from the Academic Editor

In addition to comments from the reviewer about overuse/interpretation of P-value, I also agree that further discussion of the systematic error will be to the benefit of this paper.

Thank for the observation, we have made the necessary correction throughout the manuscript.

Review Comments to the Author

Please use the space provided to explain your answers to the questions above. You may also include additional comments for the author, including concerns about dual publication, research ethics, or publication ethics. (Please upload your review as an attachment if it exceeds 20,000 characters)

**Reviewer #1:**

I thank the authors for their revisions. I have a few minor comments that I believe will help improve the manuscript.

Line 48: MTCT is no longer abbreviated in the manuscript and therefore should be written out.

Thank you, MTCT has been written in full in the manuscript.

Lines 108-112: While the information is available in the tables, it may be useful to include the categories of responses in the text. Something along the lines of "HIV status of spouse (yes; no)".

The following sentence has been inserted: “Concerning HIV status of spouses, 4.2% of HIV negative women had HIV positive partners while 29.6% of HIV positive women had an HIV negative partner.”

Line 133: Only means are mentioned in the edited version but standard deviations are still calculated and presented in tables.

This has been corrected.

Lines 136-137: I see that the authors removed most of the p-values as previously suggested. Therefore, I am not sure why statistical tests (Chi-square and t-test/rank-sum) are discussed in the analysis section. Additionally, the authors may want to mention the use of the Kaplan-Meier estimator to generate the survival curves.

This has been corrected and the paragraph now reads as follows: “HIV-free survival was estimated as the proportion of children alive and HIV-negative among all exposed children. The precision around survival estimates was assessed by 95% confidence intervals. We used the Kaplan Meier curves to graphically display infant mortality, infection, and HIV free survival. We performed complete case analysis, and missing data were not imputed.”

Lines 166, 168, 202: It appears that p-values are still used for interpreting some descriptive results.

This has been corrected.

Lines 239, 240, 242, 283: The word "significant" is used to describe the results. While maybe not what the authors intend, this may easily be misread by readers as statistically significant, so I would suggest replacing or removing the word "significant".

The language has been corrected.

Lines 302-312: While the authors did add to their limitations, I believe that further discussion of systematic errors such as measurement error would be beneficial.

Thank you. We have added additional explanation.

Reviewer #2: Minor comment: Line 26 in the abstract, the authors conclude that “Implementation of universal maternal ART lowers MTCT BY 6 weeks of age ...”. This conclusion is not supported by the findings. Did you mean “at 6 weeks of age? as is consistent with the conclusion in the main paper (line 288)

Thank you. The sentence in the abstract has been corrected to align with the conclusion of the manuscript.

Reviewer #3: Authors have addressed my initial comments adequately and I have no additional comments. Recommend manuscript for publication.

Thank you very much.
